# Osseointegration of Plasma Jet Treated Titanium Implant Surface in an Animal Model

**DOI:** 10.3390/ma14081942

**Published:** 2021-04-13

**Authors:** Min-Ho Jang, Young-Bum Park, Jae-Sung Kwon, Yeun-Ju Kim, Jae-Hoon Lee

**Affiliations:** 1 Department of Prosthodontics, Yonsei University College of Dentistry, Seoul 03722, Korea; jmh0308@naver.com (M.-H.J.); drybpark@yuhs.ac (Y.-B.P.); yeunju@yuhs.ac (Y.-J.K.); 2 Department and Research Institute of Dental Biomaterials and Bioengineering, Yonsei University College of Dentistry, Seoul 03722, Korea; jkwon@yuhs.ac; 3 BK21 FOUR Project, Yonsei University College of Dentistry, Seoul 03722, Korea

**Keywords:** implant, non-thermal atmospheric pressure plasma jet (NTAPPJ), osseointegration, implant surface, titanium implant, animal study

## Abstract

Osseointegration of titanium implant is important for the success of both dental and medical implants. Previous studies have attempted to improve osseointegration by considering the use of plasma jet technology, where information with animal models and parameters related to osseointegration is still lacking. Therefore, this study investigated the effects of non-thermal atmospheric pressure plasma jet (NTAPPJ) treatment on titanium implants in terms of osseointegration in mongrel dogs. A total of 41 implants; 21 NTAPPJ treated and 20 control, were placed in the maxilla and mandible of six mongrel dogs for either 4 or 8 weeks. The bone volume (BV) and bone-to-implant contact (BIC) ratio were determined by region of interest (ROI). Statistical analysis was performed with the Wilcoxon rank-sum test. The NTAPPJ group at 4 weeks showed higher numbers in both BV and BIC (*p* < 0.05) compared to the control group. However, at 8 weeks there were less significant differences between the control or experimental group as the control group had caught up with the experimental group. Hence, NTAPPJ may be an effective treatment for the initial healing period which is critical to ensure reliable long-term predictability. The BV and BIC have been clinically proven to accelerate in the initial stages with the use of NTAPPJ to aid in the healing and initial stability of implants.

## 1. Introduction

Titanium alloys are commonly used as implant material due to their superior mechanical property and biocompatibility [1]. Clinical success being critically dependent on osseointegration between the titanium implant and the living bone, various surface treatments are currently under development to ensure and strengthen the initial functional connection between the implant and the living bone [2]. Osseointegration has been defined as a direct and functional connection between bone and an artificial implant [3]. Sandblasted acid-etching (SA, blasting with Al_2_O_3_, TiO_2_, TiO_3,_ or Ca_3_PO_4_ resorbable media) and hydroxyapatite coating are among the more widely used methods [4,5]. Several studies have shown that these methods yield better bone-to-implant contact (BIC) than do machined implant surfaces [6,7,8]. Nevertheless, it is difficult to exceed a BIC of 50%, far from the ideal 100% [9,10].

The phenomenon known as biological aging, by which surface properties of implants tend to change significantly over time, is gaining recognition as a possible explanation for the less than ideal BIC of titanium implants. After 4 weeks of storage in an ambient condition, the percentage of carbon element increased from 20% to 63% [11,12]. The ability of the titanium surface to attract proteins and osteogenic cells is thought to be inversely correlated with the percentage of surface carbon, which will eventually cause degradation of the biological activity of titanium implant [11]. 

There have been numerous efforts to find a method to overcome biological aging. For example, UV treatment removes oxygen-containing hydrocarbons covering the TiO_2_ surface [13]. This treatment also makes the surface super-hydrophilic by increasing the surface energy of the surface of TiO_2_ through photocatalysis [14]. Another surface treatment with an effect similar to UV treatment is non-thermal atmospheric pressure plasma jet (NTAPPJ), which decomposes and removes chemical contamination of hydrocarbons from titanium surfaces [15]. A previous study that considered the application of NTAPPJ with exactly the same configuration as this study on an SA surface of titanium indicated reduction of hydrocarbon related peak in X-ray photoelectron spectroscopy (XPS) analyses along with radically reducing the contact angle, and thus increasing the wettability of implants by increasing surface energy [15]. Such findings were also concurred by another study group that considered use of NTAPPJ on titanium implant [16]. The absence of hydrocarbons and reduced contact angle on the implant surface increases the absorption of blood proteins such as serum albumin or plasma fibronectin by inducing adhesion and growth of osteoblasts [15,16]. The percentage of hydrocarbons and the hydrophilic property thus play an important role in implant biocompatibility due to their effect on implant–protein–cell interaction [15,16,17,18,19,20]. UV treatment and NTAPPJ are similar in both their application method and effects on implants, therefore future NTAPPJ studies can build onto existing UV treatment research.

NTAPPJ is an electrically neutral, ionized gas under normal pressure conditions that alters surface energy and chemistry by generating a high concentration of reactive species. This method differs from the thermal plasma treatment traditionally used with hydroxyapatite coatings on implant surfaces (plasma spraying). NTAPPJ is a much cheaper and easier process to induce chemical changes on the implant surface, rather than the thermal plasma process that requires a vacuum chamber [15].

Previous studies on the biological effect of NTAPPJ on commercial implants are limited in number and no in vivo studies have been conducted [21,22,23]. In most of these studies, researchers measured only bone-to-implant contact (BIC) cross-sectionally, which does not reflect the overall new bone formation around implants. To resolve this limitation, this study additionally investigated new bone generation by measuring bone volume (BV) around the implant in three dimensions; using these results to determine whether the NTAPPJ treatment on an SA surface implant can improve osseointegration in dogs during different healing periods.

## 2. Materials and Methods

### 2.1. Animal Model

Implants were placed in the maxilla and mandible of six mongrel dogs; all specimens were healthy and in good nutrition with no periodontal disease such as gingivitis or periodontitis. This experiment was conducted following the standard protocol defined by the Laboratory Animal Management Committee of Medical College at Yonsei University (2011-0306). All sections of this report adhere to the ARRIVE Guidelines for reporting animal research [24]. 

### 2.2. Titanium Implants

**Figure 1:**
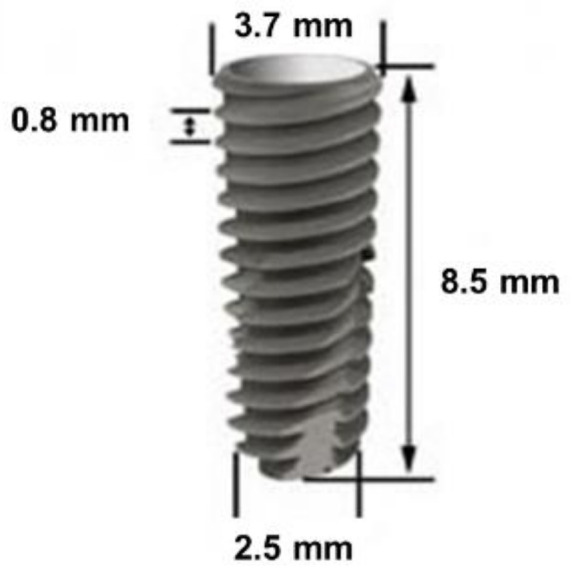
Design of implant fixture used in this study.

A total of 41 SA surface implants Ø3.5 mm × 8.5 mm in size (TSIII SA fixture, Osstem implant system, Busan, Korea) were used for this experiment (Figure 1). Plasma treatment was conducted on 20 implants in the experimental group whereas the remaining non-treated 21 implants were used as the control group. 

### 2.3. Treatment by Non-Thermal Atmospheric Pressure Plasma Jet

**Figure 2:**
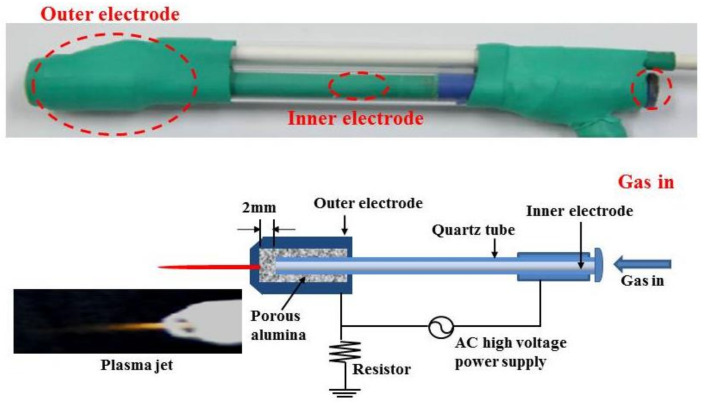
Schematic drawing of non-thermal atmospheric pressure plasma jet provided by Plasma Bioscience Research Center at Kwangwoon University.

The experimental groups were exposed to NTAPPJ, 2 h prior to implantation. The NTAPPJ device was adapted from the Plasma Bioscience Research Center at Kwangwoon University (Seoul, Korea) (Figure 2). All experiments were carried out with a nitrogen gas flow of 5 L/min and a flume end-to-sample distance set to 3 mm (max output voltage 15 kV, current 13 mA). Implants in the experimental group were treated with plasma for 10 min.

### 2.4. Surface Morphology and Chemical Characterization 

The surface morphology was observed by scanning electron microscope (SEM; Carl Zeiss, Oberkochen, Germany) at 20 kV and 5000× magnification, before and after NPAPPJ exposure. The surface chemical characterization was determined by X-ray photoelectron spectroscopy (XPS; K-alpha, Thermo VG Scientific, Waltham, MA, USA), before and after 10 min of NTAPPJ exposure as above but with additional analysis with 2 min of NPAPPJ exposure in order to understand the trend of chemical changes. Monochromatic Al K was used as the X-ray source (Al K line: 1486.6 eV). The spectra were recorded with a pass energy of 200 eV (step size of 1.0 eV) in survey mode and 50 eV (step size of 0.1 eV) in the high-resolution mode to acquire the C1s and O1s spectra with a resolution of 0.78 eV measured from the Ag 3d5/2 peaks. The binding energies were referenced to the C1s peak at 284.8 eV.

### 2.5. Surgical Protocol on Animal Model

**Figure 3:**
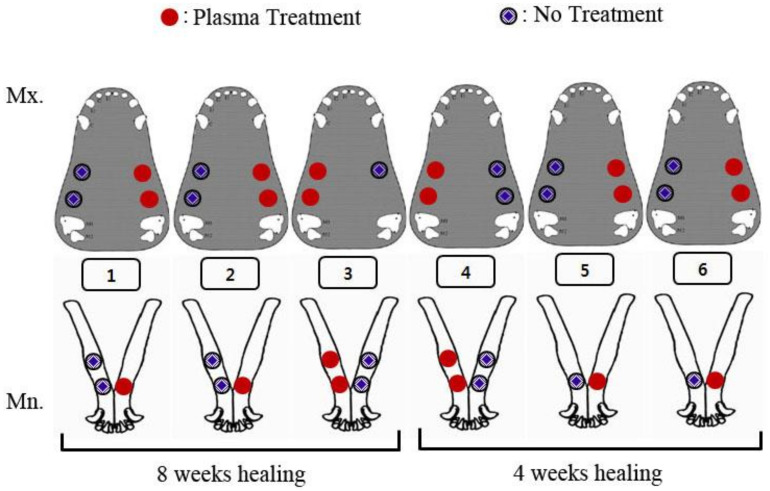
Schematic diagram of experimental design.

A total of 41 implants was divided into two groups (control and experimental); 21 non-plasma treated SA implants were used as the control group and 20 plasma treated SA implants were used as the experimental group. Three and four implants were placed in the maxilla and the mandible, respectively. All the implants in both groups were divided into two subgroups: one group was sacrificed at 4 weeks after implantation and another group at 8 weeks after implantation (Figure 3). With the dogs under general anesthesia in a sterilized environment, the premolars and the first molar were extracted from the maxilla and the mandible. Two months after the extraction, a crestal incision and full mucoperiosteal flap were made and the implants were placed in the maxilla and the mandible under the same conditions. One or two implants were placed in each quadrant of the maxilla and the mandible, respectively. Every step of implantation complied with the manufacturer’s recommendations. Post-operative management was conducted similar to the post-extraction management.

### 2.6. Histomorphometric Analysis

For the histomorphometric analysis, the amount of bone around the implant was calculated as bone volume/total volume (BV/TV), and the bone-to-implant contact (BIC) was measured for outcome in the region of interest (ROI). Bone volume was analyzed in a cylindrical shape defined by 3 best threads and a circumferential zone within 50 µm of the implant surface. A 3-dimensional bone volume analysis was conducted using micro-computed tomography (micro-CT) (SkyScan 1173, Bruker, Billerica, MA, USA). Micro-CT uses X-rays to create cross-sections of a physical object, which are used to recreate a virtual 3D model, which allows no damage to the original object. Bone volume data were calculated with CTVol (v.2.2) software (Bruker, Billerica, MA, USA) and the bone-to-implant contact (BIC) ratio was expressed as the amount of bone that contacts the implant surface directly along with the ROI which was manually defined [25] and was measured with i-Solution software (Ver. 11.0, IMT i-Solution Inc., Burnaby, BC, Canada).

### 2.7. Statistical Analysis

Means and standard deviations (SD) of all obtained values were calculated for each group for bone volume (BV) and bone-to-implant contact (BIC). The Wilcoxon rank-sum test was used to calculate the significance of the differences in bone volume (BV) and BIC between groups. The level of statistical significance was set at *p* < 0.05. The outlier samples (those not included within the 2SD range) were excluded from statistical analysis with bone volume (BV). Additionally, a 2-way analysis of variance (ANOVA) was used to consider the times (4 weeks and 8 weeks) and groups (experimental and control). Data were analyzed using the SPSS 25 software program (IBM Corp., Armonk, NY, USA) at the 0.05 level of significance.

## 3. Results

### 3.1. Surface Morphology

**Figure 4:**
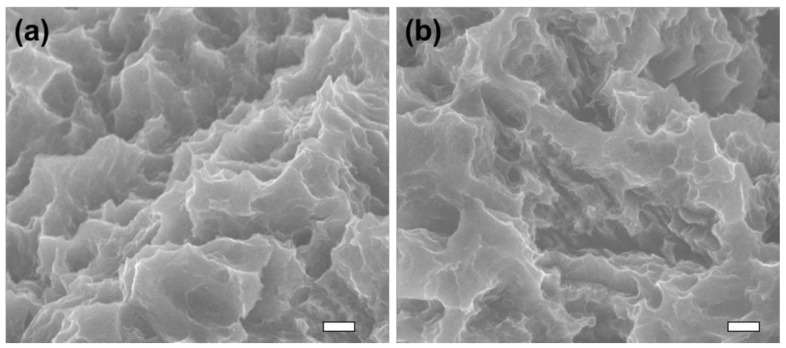
Scanning electron microscopy images of titanium implant before (**a**) and after (**b**) non-thermal atmospheric pressure plasma jet (NTAPPJ) treatment. Scale bar is 2 µm.

The results of surface morphology examination with SEM are shown in Figure 4. The results showed the typical rough surface of SA titanium. There were no evident changes before and after NTAPPJ treatment in terms of the morphology.

### 3.2. Surface Chemistry 

**Figure 5:**
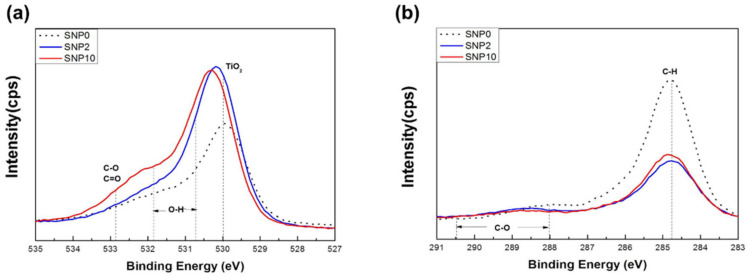
X-ray photoelectron spectroscopy (XPS) analyses on titanium surface before NTAPPJ treatment (SNP0), following 2 min of NTAPPJ treatment (SNP2) and following 10 min of NTAPPJ treatment (SNP10) with detailed spectra for O1s (**a**) and C1s (**b**).

The results of surface chemical characterization with XPS are shown in Figure 5. The results from O1s spectra (Figure 5a) indicated a general increase in peak intensity relevant to TiO_2_ with a larger area under the curve following exposure of NTAPPJ. With longer duration of exposure, peaks corresponding to C-O, C=O, and O-H increased. In terms of C1s spectra (Figure 5b), a dramatic decrease in C-H peak with a smaller area under the curve was evident following exposure to NTAPPJ. There were no changes observed with longer duration of exposure to NTAPPJ.

### 3.3. Histomorphometric Analysis

The experimental group showed a statistically significant (*p* < 0.05) higher bone volume (BV) than the control group at 4 weeks. The mean BV at 4 weeks was 57.88% (SD: 6.52) in the experimental group vs 49.20% (SD: 6.83) in the control group (Table 1). The mean bone volume of the experimental group (63.2%) was higher than that of the control group (62.14%) at 8 weeks as well but showed no statistically significant difference. 

The histomorphometric data of BIC obtained from the three-thread length revealed the same tendency with BV (Table 1). The mean BIC of the experimental group was 80.9 (SD: 9.85) and the control group was 70.85 (SD: 17.65) at 4 weeks which was significantly different (*p* < 0.05). The mean value of BIC of the experimental group (81.9%) was higher than the control group (77.95%) at 8 weeks but was not statistically significant.

Differences at 4 weeks and 8 weeks and between experimental and control groups were analyzed using a 2-way analysis of variance (ANOVA). The result of the 2-way ANOVA with BV was found to be significant between different times and groups, BIC showing significant differences between experimental and control groups.

## 4. Discussion

Chemical and biological properties of the titanium surface were found to change over time, biological aging of titanium is due to increased surface carbon [11,26,27]. During this process, hydrocarbon and cations make the TiO_2_ surface electronegative at the physiologic pH value [28,29]. However, Wael Att et al. proved that a freshly exposed titanium surface is electropositive [11]. Serum albumin molecules that directly contact the titanium surface upon surgery are known to be electronegative, making the new titanium surface a chemoattractant for proteins. Enhanced protein adsorption should lead to enhanced cell attachment as cell–protein interaction increases via ligand-specific binding. It is noteworthy that the electropositive surface of the newly processed titanium allows not only proteins but also cells to directly attach to the surface [11]. Unfortunately, TiO_2_ undergoes additional changes once surrounding ions and carbon compounds bind to its surface. The electropositive surface thus becomes electronegative and only attracts proteins with divalent cations such as Ca^2+^, yielding a decreased binding affinity between the old TiO_2_ surface and the proteins [28].

NTAPPJ is a process to remove hydrocarbon from the TiO_2_ surface, making the surface electropositive and thus restoring its protein and cell-attractive property. NTAPPJ produces plasma at low-temperature under atmospheric pressure. When voltage is applied to the gas flowing between the electrode, an ionized gas and a chemically reactive medium is formed. The ability of NTAPPJ to decrease hydrocarbon on the TiO_2_ surface has been previously confirmed by X-ray photoelectron spectroscopy (XPS). Lee et al. analyzed the chemical composition of atmospheric pressure plasma-treated surfaces and non-treated surfaces using XPS [15]. Their analysis showed that plasma jet treatment reduced the proportion of hydrocarbon as well as overall oxidization with an increase in O-H, C-O, or C=O groups [15,30]. This result was in agreement with our analyses of NTAPPJ treated SA surface titanium, as C-H peak reduces corresponding with reduction of hydrocarbon, along with an increase in O-H, C-O and C=O peaks in O1s spectra for NTAPPJ treated SA titanium surface. 

Although histological imaging is still considered the gold standard for analyzing bone formation around implants, we used micro-CT to analyze bone volume in this study. As only a few histologic slide images can be obtained from a bone specimen, the amount of data obtained from the slides is limited. Moreover, the histomorphometric method cannot reflect the overall new bone formation around the fixture after implantation. The purpose of true bone volume analysis is to calculate the amount of new bone around a fixture. The only shortcoming of micro-CT is the generation of artifacts around the fixture. Song et al. reported that despite the limitations to measure the BV correctly due to such artifacts, correlation with tissue slides facilitated valid bone morphometry by micro-CT [31]. They also noted that the micro-CT indicated a greater mean bone volume than that of the tissue slide. Bone volume data obtained in this experiment were thus assumed to be higher than that of the actual value. This did not, however, substantially affect the relative values of the experimental and control groups. In order to eliminate type I error, four samples at 4 weeks and one sample at 8 weeks were excluded from the statistical analysis because the mean bone volume was out of the 2SD range. Two of the 4 week control group samples were excluded because they showed a highly irregular bone volume pattern.

Hideki Aita et al. used bone volume analysis to investigate whether UV treatment of titanium enhanced osteoconductive capacity [13]. They found the bone volume in the 50 µm zone around the fixture surface in UV treated groups was significantly greater than that in control groups. Based on this finding, we decided to analyze bone volume within 50 µm of the implant surface. We found a difference in bone levels when observing coronal slide views sectionalized at the midpoint of the fixtures. In other words, marginal bone was seen on the other thread of fixtures in the coronal section view of micro-CT. This was due to uneven bone levels around fixtures installed in alveolar bones with varying morphologies. We thus used the best thread technique to obtain the largest values for bone volume area in the fixtures.

UV treatment has an effect similar to that of NTAPPJ. Previous studies have found that UV treatment changed the titanium surface from hydrophobic to super-hydrophilic [14]. When an implant surface becomes hydrophilic due to high surface energy, the interaction between protein, implant, and cells improves, increasing implant bio-compatibility [15,16,17,18,19,20]. NTAPPJ effectively increases wettability of metal, ceramic, and polymer surfaces [32,33]. NTAPPJ treatment of a titanium implant generates high surface energy, making the surface hydrophilic. This chemical change in the titanium surface increases attachment and proliferation of osteoblast cells, which in turn enhances cellular activity on the titanium implant surface [15,16]. As the implant surface directly contacts blood and extracellular matrixes after implantation, its hydrophilic nature plays a critical role in osseointegration.

A statistically significant difference between the 4 week groups suggests that NTAPPJ affects bone–implant integration at an early stage when stability is crucial for immediate loading. From this study, we found that NTAPPJ can affect early bone formation, which is 4 weeks after implantation, which correlates with results from previous NTAPPJ studies [21,22,23]. The results of these studies are summarized in Table 2. 

In addition, various in vitro models were also considered for possibilities of using non-thermal plasma treated dental implant. Kathrin Duske et al. showed that NTAPPJ reduced the contact angle and assisted the spread of osteoblastic cells [16]. Kwon et al. found significantly improved osteoblast attachment with a relatively shorter duration of NTAPPJ [34].

Treatment with NTAPPJ has many advantages. Being simple, inexpensive, easy to use, and time efficient, it has potential for routine clinical use. The results of this study indicate that plasma treatment of the titanium surface before implantation increases implant viability. Increased bone–implant integration through NTAPPJ may result in consistent and predictable implantation. Other issues affecting implant prognosis, including cytotoxicity, remain to be investigated. Additional animal experiments and clinical research with NTAPPJ under various conditions are warranted.

## 5. Conclusions

Despite the limitations of animal study, NTAPPJ seems to enhance the process of bone–implant integration in the initial healing stage, which could possibly explain that NTAPPJ enhanced the time dependent degradation of the titanium implant. Based on these results, previously reported evidences, and providing that additional studies would be carried out, it would be possible to deduce the conclusion that NTAPPJ may be an effective and practical treatment where initial stability of implant and healing are more demanding ensuring reliable long-term predictability.

## Figures and Tables

**Table 1:** Mean ± standard deviations of bone volume (BV) and bone-to-implant contact (BIC) ratio.

Duration of Implant and Groups		BV (Mean ± SD, %)	BIC (Mean ± SD, %)
4 weeks	Experimental group	57.88 ± 6.52 *	80.9 ± 9.85 *
	Control group	49.20 ± 6.83 *	70.85 ± 17.65 *
8 weeks	Experimental group	63.20 ± 11.28	81.9 ± 15.92
	Control group	62.14 ± 10.20	77.95 ± 15.18

* Significant differences between experimental and control group (*p* < 0.05).

**Table 2:** Summary of previous studies that considered effects of non-thermal plasma on titanium implant in animal models.

Author Name	Type and Mode of Non-Thermal Plasma Used	Animal Model Used	Parameter(s) Considered	Major Findings
Teixeira, et al. [21]	KinPen^TM^ device^1^ for either 20 s or 60 s exposure	Radius diaphysis of beagle dogs	Removal torque of implants in 2 and 4 weeks	Significantly increased torque values following plasma exposure
Zheng, et al. [22]	CPActive device^2^ with argon gas flow	Maxillary first molar site of Sprague Dawley rats	BIC, BV, trabecular thickness, and trabecular separation at 2 to 6 weeks of implant	25% to 40% increase in BIC
Hung, et al. [23]	Dielectric barrier discharge with argon flow of 1.8 L/min and oxygen flow of 0.01 L/min^3^	Jawbone of beagle dogs	Implant stability quotient (ISQ) following 4, 8, and 12 weeks of implant	Increased the healing time slightly during the early recovery period

^1^ Commercially available device from INP-Greifswald, Greifswald, Germany. ^2^ Apparatus manufactured by Chengdu, China. ^3^ Apparatus manufactured by Yih Dar Technology, Hsinchu County, Taiwan.

## Data Availability

The datasets used and/or analyzed during the current study are available from the corresponding author on reasonable request.

## Summary of Abbreviations

NTAPPJ	Non-Thermal Atmospheric Pressure Plasma Jet
SA	Sandblasted Acid-etching
BIC	Bone-to-Implant Contact
BV	Bone Volume
TV	Bone Volume
ROI	Region Of Interest

## References

[1] Lemons J.E., Niemann K.M.W., Weiss A.B. (1976). Biocompatibility studies on surgical-grade titanium-, cobalt-, and iron-base alloys. J. Biomed. Mater. Res..

[2] Albrektsson T., Brånemark P.-I., Hansson H.-A., Lindström J. (1981). Osseointegrated titanium implants: Requirements for ensuring a long-lasting, direct bone-to-implant anchorage in man. Acta Orthop. Scand..

[3] Giudice A., Bennardo F., Antonelli A., Barone S., Wagner F., Fortunato L., Traxler H. (2020). Influence of clinician’s skill on primary implant stability with conventional and piezoelectric preparation techniques: An ex-vivo study. J. Biol. Regul. Homeost. Agents.

[4] Albrektsson T., Wennerberg A. (2004). Oral implant surfaces: Part 1—Review focusing on topographic and chemical properties of different surfaces and in vivo responses to them. Int. J. Prosthodont..

[5] Albrektsson T., Wennerberg A. (2004). Oral implant surfaces: Part 2—Review focusing on clinical knowledge of different surfaces. Int. J. Prosthodont..

[6] Wennerberg A., Albrektsson T. (2010). On implant surfaces: A review of current knowledge and opinions. Int. J. Oral Maxillofac. Implant..

[7] Wennerberg A., Hallgren C., Johansson C., Danelli S. (1998). A histomorphometric evaluation of screw-shaped implants each prepared with two surface roughnesses. Clin. Oral Implant. Res..

[8] Ogawa T., Ozawa S., Shih J.-H., Ryu K., Sukotjo C., Yang J.-M., Nishimura I. (2000). Biomechanical Evaluation of Osseous Implants Having Different Surface Topographies in Rats. J. Dent. Res..

[9] Weinlaender M., Kenney E.B., Lekovic V., Beumer J., Moy P.K., Lewis S. (1992). Histomorphometry of bone apposition around three types of endosseous dental implants. Int. J. Oral Maxillofac. Implant..

[10] Ogawa T., Nishimura I. (2003). Different bone integration profiles of turned and acid-etched implants associated with modulated expression of extracellular matrix genes. Int. J. Oral Maxillofac. Implant..

[11] Att W., Hori N., Takeuchi M., Ouyang J., Yang Y., Anpo M., Ogawa T. (2009). Time-dependent degradation of titanium osteoconductivity: An implication of biological aging of implant materials. Biomaterials.

[12] Att W., Hori N., Iwasa F., Yamada M., Ueno T., Ogawa T. (2009). The effect of UV-photofunctionalization on the time-related bioactivity of titanium and chromium–cobalt alloys. Biomaterials.

[13] Aita H., Hori N., Takeuchi M., Suzuki T., Yamada M., Anpo M., Ogawa T. (2009). The effect of ultraviolet functionalization of titanium on integration with bone. Biomaterials.

[14] Wang R., Hashimoto K., Fujishima A., Chikuni M., Kojima E., Kitamura A., Shimohigoshi M., Watanabe T. (1997). Light-induced amphiphilic surfaces. Nat. Cell Biol..

[15] Lee E.-J., Kwon J.-S., Uhm S.-H., Song D.-H., Kim Y.H., Choi E.H., Kim K.-N. (2013). The effects of non-thermal atmospheric pressure plasma jet on cellular activity at SLA-treated titanium surfaces. Curr. Appl. Phys..

[16] Duske K., Koban I., Kindel E., Schröder K., Nebe B., Holtfreter B., Jablonowski L., Weltmann K.D., Kocher T. (2012). Atmospheric plasma enhances wettability and cell spreading on dental implant metals. J. Clin. Periodontol..

[17] Lang N.P., Salvi G.E., Huynh-Ba G., Ivanovski S., Donos N., Bosshardt D.D. (2011). Early osseointegration to hydrophilic and hydrophobic implant surfaces in humans. Clin. Oral Implant. Res..

[18] Zhang Y., Andrukhov O., Berner S., Matejka M., Wieland M., Rausch-Fan X., Schedle A. (2010). Osteogenic properties of hydrophilic and hydrophobic titanium surfaces evaluated with osteoblast-like cells (MG63) in coculture with human umbilical vein endothelial cells (HUVEC). Dent. Mater..

[19] Ivanovski S., Hamlet S., Salvi G., Huynh-Ba G., Bosshardt D., Lang N.P., Donos N. (2011). Transcriptional profiling of osseointegration in humans. Clin. Oral Implant. Res..

[20] Rupp F., Scheideler L., Olshanska N., De Wild M., Wieland M., Geis-Gerstorfer J. (2006). Enhancing surface free energy and hydrophilicity through chemical modification of microstructured titanium implant surfaces. J. Biomed. Mater. Res. Part A.

[21] Teixeira H.S., Marin C., Witek L., Freitas A., Silva N.R., Lilin T., Tovar N., Janal M.N., Coelho P.G. (2012). Assessment of a chair-side argon-based non-thermal plasma treatment on the surface characteristics and integration of dental implants with textured surfaces. J. Mech. Behav. Biomed. Mater..

[22] Zheng Z., Ao X., Xie P., Wu J., Dong Y., Yu D., Wang J., Zhu Z., Xu H.H.K., Chen W. (2020). Effects of novel non-thermal atmospheric plasma treatment of titanium on physical and biological improvements and in vivo osseointegration in rats. Sci. Rep..

[23] Hung Y.W., Chen H.L., Lee L.T., Tung K.C., Bau D.T., Wong Y.K. (2018). Effects of non-thermal plasma on sandblasted titanium dental implants in beagle dogs. J. Chin. Med. Assoc..

[24] Kilkenny C., Browne W.J., Cuthill I.C., Emerson M., Altman D.G. (2010). Improving bioscience research reporting: The ARRIVE guidelines for reporting animal research. PLoS Biol..

[25] Tonino A.J., Thèrin M., Doyle C. (1999). Hydroxyapatite-coated femoral stems. Histology and histomorphometry around five components retrieved at post mortem. J. Bone Joint. Surg. Br..

[26] Hori N., Att W., Ueno T., Sato N., Yamada M., Saruwatari L., Suzuki T., Ogawa T. (2009). Age-dependent Degradation of the Protein Adsorption Capacity of Titanium. J. Dent. Res..

[27] Iwasa F., Hori N., Ueno T., Minamikawa H., Yamada M., Ogawa T. (2010). Enhancement of osteoblast adhesion to UV-photofunctionalized titanium via an electrostatic mechanism. Biomaterials.

[28] Ellingsen J.E. (1991). A study on the mechanism of protein adsorption to TiO_2_. Biomaterials.

[29] Klinger A., Steinberg D., Kohavi D., Sela M. (1997). Mechanism of adsorption of human albumin to titanium in vitro. J. Biomed. Mater. Res. Part A.

[30] Cho S., Jung C. (2010). Surface modification of TiO₂ by atmospheric pressure plasma. J. Korean Vac. Soc..

[31] Song J.W., Cha J.Y., Bechtold T.E., Park Y.C. (2013). Influence of peri-implant artifacts on bone morphometric analysis with micro-computed tomography. Int. J. Oral Maxillofac. Implant..

[32] Han I., Vagaska B., Park B.J., Lee M.H., Lee S.J., Park J.-C. (2011). Selective fibronectin adsorption against albumin and enhanced stem cell attachment on helium atmospheric pressure glow discharge treated titanium. J. Korean Vac. Soc..

[33] Koban I., Duske K., Jablonowski L., Schröder K., Nebe B., Sietmann R., Weltmann K.-D., Hübner N.-O., Kramer A., Kocher T. (2011). Atmospheric Plasma Enhances Wettability and Osteoblast Spreading on Dentin In Vitro: Proof-of-Principle. Plasma Process. Polym..

[34] Kwon J.-S., Kim Y.H., Choi E.H., Kim K.-N. (2013). The effects of non-thermal atmospheric pressure plasma jet on attachment of osteoblast. Curr. Appl. Phys..

